# Intravenous immunoglobulin in the therapy of adult acute fulminant myocarditis: A retrospective study

**DOI:** 10.3892/etm.2013.1372

**Published:** 2013-10-29

**Authors:** DAN-QING YU, YING WANG, GUI-ZHOU MA, RONG-HE XU, ZHI-XIONG CAI, CHU-MIN NI, PING CHEN, ZHI-DAN ZHU

**Affiliations:** 1 Department of Coronary Care Unit, Guangdong Cardiovascular Institute, Guangdong Academy of Medical Science, Guangdong General Hospital, Guangzhou, Guangdong 510080, P.R. China; 2 Department of Cardiology, Affiliated Shantou Hospital of Sun Yat-sen University, Jinping, Shantou, Guangdong 515031, P.R. China

**Keywords:** acute fulminant myocarditis, heart failure, intravenous immunoglobulin, left ventricular ejection fraction, arrhythmia

## Abstract

Acute fulminant myocarditis (AFM) is a serious heart disease with limited treatment. This observational retrospective study aimed to investigate whether intravenous immunoglobulin (IVIG) was able to improve left ventricular function and reduce the episodes of arrhythmia in adult patients with AFM. The medical records of all patients with AFM who were admitted to the Critical Care Unit of Guangdong General Hospital (Guangzhou, China) between January 2001 and December 2010 were reviewed. A cohort of 58 patients was included in the study. Of these 58, 32 patients were treated with IVIG (400 mg/kg per day) for five days, while the remaining patients did not receive IVIG therapy. The patients who received IVIG therapy had a higher left ventricular ejection fraction (LVEF) and a reduced left ventricular end-diastolic diameter (LVDD) compared with the non-IVIG therapy patients four weeks subsequent to the treatment (P_LVEF_=0.011 and P_LVDD_=0.048). The post-treatment incidence of ventricular tachycardia/ventricular fibrillation (VT/VF) and atrioventricular block (AVB) was reduced in the patients who received IVIG therapy compared with the baseline values (P_VT/VF_=0.025, P_AVB_=0.003); however, no significant differences were observed in the non-IVIG therapy patients (P_VT/VF_=0.564, P_AVB_=0.083) following treatment. There were two mortalities in the IVIG therapy group and seven in the non-IVIG therapy group (P=0.072). This retrospective study suggested that the use of IVIG for the treatment of AFM may be associated with improved left ventricular function and reduced episodes of fulminant arrhythmias.

## Introduction

Acute fulminant myocarditis (AFM) is clinically and pathologically defined as inflammation of the myocardium, leading to the sudden onset of heart failure, arrhythmia, fulminant hemodynamic collapse and sudden mortality (1,2). The initial severe presentation and favorable long-term prognosis of AFM is associated with left ventricular function (3). AFM involves serious arrhythmias, which occasionally have lethal consequences due to cardiac dysfunction (4).

Intravenous immunoglobulin (IVIG) has been used to treat inflammatory and autoimmune diseases (5,6). Immune or autoimmune mechanisms may be involved in the pathogenesis of myocarditis (7). The administration of immunoglobulin was reported to be of clinical value against certain viral infections due to the neutralization of the virus, the blockade of Fc receptors and the neutralization of a microbial toxin (5,6,8,9). Although the immunosuppressive therapy has yielded conflicting results in patients with acute myocarditis (10), previous studies demonstrated the effect of the IVIG in acute myocarditis (9,11). The beneficial effect of IVIG on myocarditis was demonstrated in an animal study using polyclonal immunoglobulin (11). In the pediatric population, IVIG was associated with improved recovery of left ventricular function (9). However, few studies have specifically focused on IVIG for adult AFM. This retrospective study was performed to evaluate the effect of IVIG on the cardiac function and cardiac rhythm of adult patients with AFM.

## Methods

### Patients and study design

This was an observational retrospective case study of inpatients who presented with AFM in Guangdong General Hospital (Guangzhou, China) between January 2001 and December 2010. The patients were included according to the following criteria: Adult (age >18 years), acute-onset (duration <3 months) congestive heart failure and impaired left ventricular function following a recent viral illness. Patients with coronary artery disease, long-standing dilated cardiomyopathy, structural heart disease, systemic autoimmune disease, Kawasaki disease, the presence of active infection and other specific causes of acute cardiomyopathy were excluded. Data were collected through patient chart review. This study was approved by the ethics committee for clinical investigations of Guangdong General Hospital and informed consent was obtained from the patient’s family.

### Data collection

Clinical data and demographic information were collected by the review of the medical records of the enrolled patients. The patients were divided into IVIG therapy and non-IVIG therapy (control) groups. Blood samples were obtained to analyze myocardial enzymes, troponin, brain natriuretic peptide, C-reactive protein and erythrocyte sedimentation rate at baseline. Echocardiography, electrocardiography and 24-h ambulatory electrocardiography were performed prior to and following treatment. Gender, age, cardiac function classification, parameters of echocardiography, blood test data and incidence of complications were compared between the two groups.

### Echocardiography

Echocardiography data were collected by previously reported methods (12). Left ventricular ejection fraction (LVEF), diameter of the left atrium (LA), left ventricular end-diastolic diameter (LVDD), left ventricular systolic diameter (LVDS), diameter of the right atrium (RA) and diameter of the right ventricle (RV) were measured using echocardiography. The data of left ventricle and left atrium were measured on parasternal long-axis view, and the data of right ventricle and right atrium were measured on apical four-chamber view. The recovery of left ventricular function was assessed in hospital and post-treatment (four weeks).

### IVIG treatment regimens

IVIG (CSL Behring, Marburg, Germany) was administered at a dose of 400 mg/kg per day for five days. Other conventional therapies were administered as required, including high-dose vitamin C, diuretics, digoxin, dopamine, dobutamine, angiotensin-converting enzyme inhibitor/angiotensin receptor blocker (ACEI/ARB), vasodilators (sodium nitroprusside, nitroglycerin) and glucocorticoids. Intra-Aortic Balloon Pumps (IABPs) were used to treat cardiogenic shock, while temporary pacemakers were applied to third-degree atrioventricular block (AVB).

### Statistical analysis

**Figure 1:**
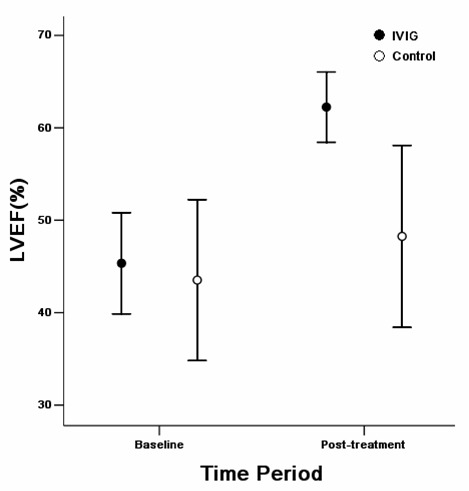
Left ventricular ejection fraction (LVEF; mean ± standard error of the mean) in the intravenous immunoglobulin (IVIG) and control groups at baseline and post-treatment (4 weeks). There was no difference between the two groups in the mean LVEF at baseline (P=0.703). Post-treatment, the LVEF of the IVIG group was higher than that of the control group (P=0.011).

The continuous variables that followed a symmetrical distribution are presented as the mean ± standard deviation, with the exception of Fig. 1. The variables in Fig. 1 are presented as the mean ± standard error. These continuous variables were compared using the two independent samples Student’s t-test. Data that followed an asymmetric distribution are presented as medians and interquartile ranges, and were compared using the Kruskal-Wallis nonparametric test. The categorical variables are presented as frequencies and were analyzed using χ^2^ and Fischer exact tests. P<0.05 was considered to indicate a statistically significant difference. All statistical analyses were performed with the Statistical Package for Social Sciences version 16.0 (SPSS Inc., Chicago, IL, USA).

## Results

### Baseline characteristics

A total of 75 patients were initially eligible for the study; however, 17 patients were excluded due to insufficient clinical data. Fifty-eight patients were ultimately included. Of these 58, 32 patients were treated with IVIG (400 mg/kg per day) for five days, while the remaining patients did not receive IVIG therapy. The IVIG and non-IVIG groups were similar with regard to baseline characteristics, including gender, age, cardiac function classification, parameters of echocardiography, blood test data and incidence of complications (Table I). The cardiac functions of the patients were classified as NYHA classes II to IV. Ten patients (33%) of the IVIG group who had NYHA class IV heart failure or cardiogenic shock received IABP support, compared with three patients (16%) in the control group (P=0.073). There were seven (27%) mortalities in the control group and two (6%) in the IVIG group (P=0.072).

### Changes in left ventricular function

**Figure 2:**
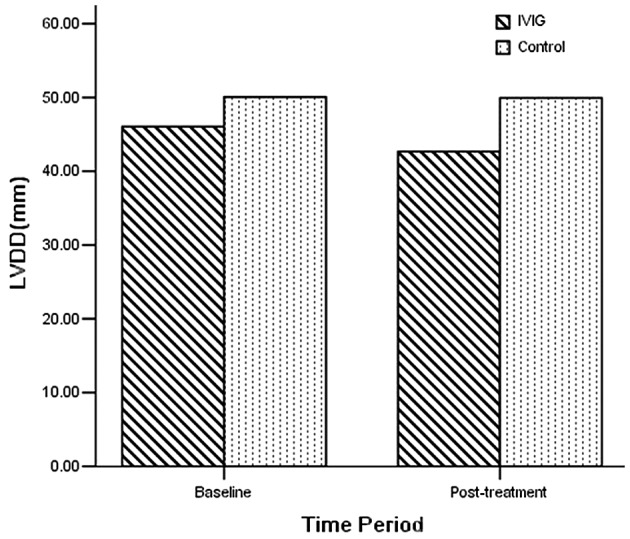
Left ventricular end-diastolic diameter (LVDD; mean ± standard deviation) in the intravenous immunoglobulin (IVIG) and control groups at baseline and post-treatment (4 weeks). There was no difference between the two groups in the mean LVDD at baseline. Four weeks subsequent to treatment, the LVDD of the IVIG group was reduced compared with that of the control group (P=0.048).

The patients of the two groups did not differ significantly with regard to the echocardiographic data at the baseline. The effect of IVIG treatment on LVEF (Fig. 1) and LVDD (Fig. 2) was investigated.

At the baseline, the LVEF of the two groups was similar (IVIG versus control group: 45.3±15.2 versus 43.5±19.6%; P=0.703). Four weeks subsequent to treatment, the mean LVEF in the IVIG group was 62.2±10.2%, compared with 48.3±20.4% in the control group. At four weeks post-treatment, the LVEF of the two groups had improved significantly compared with the baseline values (P_IVIG_<0.001, P_control_=0.027). The patients treated with IVIG had a higher LVEF than those without IVIG at four weeks (P=0.011).

**Figure 3:**
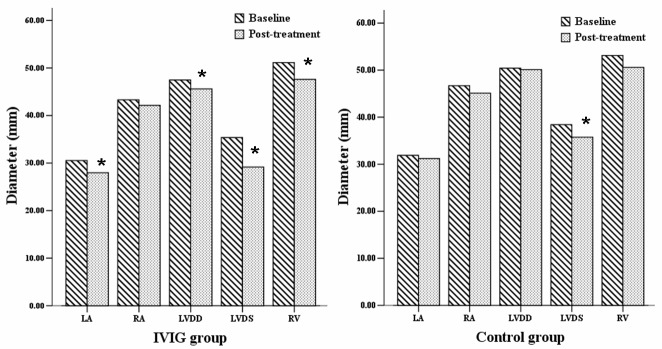
Echocardiography data (mean ± standard deviation) of the intravenous immunoglobulin (IVIG) and control groups at baseline and post-treatment (4 weeks). The diameter of the left atrium (LA), left ventricular end-diastolic diameter (LVDD), left ventricular systolic diameter (LVDS) and diameter of right ventricle (RV) of the IVIG group diminished post-treatment. The diameter of LVDS of the control group diminished. The data of left ventricle (LV) and LA were measured on parasternal long-axis view, and the data of RV and right atrium (RA) were measured on apical four-chamber view. ^*^P<0.05 vs. baseline value.

Four weeks subsequent to treatment, the LVDD of the IVIG group (44.2±5.8 mm) was reduced compared with that of the control group (49.6±10.3 mm; P=0.048; Fig. 2). Furthermore, the LA, LVDD, LVDS and RV of the IVIG group showed recovery at four weeks (P_LA_<0.001, P_LVDD_=0.006, P_LVDS_<0.001, P_RV_=0.007), with the exception of RA (P=0.232). However, only the LVDS of the control group had improved at four weeks (P_LVDS_=0.012; Fig. 3).

### Arrhythmia and changes in echocardiography results

The patients exhibited several types of arrhythmia, including bradycardia, AVB, atrial arrhythmia, ventricular arrhythmia and bundle branch block. The changes in the ST segment and T wave were also observed. Ventricular tachycardia/ventricular fibrillation (VT/VF) occurred in 8 of the 32 (25.0%) patients in the IVIG group and 3 of the 26 (11.5%) patients in the control group at baseline. Seventeen of the 32 (53.1%) patients in the IVIG group exhibited AVB at baseline compared with 8 of the 26 (30.8%) patients in the control group. Following treatment, the episodes of VT/VF and AVB were reduced in the IVIG group (P_VT/VF_=0.025, P_AVB_=0.003); however, there were no significant differences in the control group following treatment (P_VT/VF_=0.564, P_AVB_=0.083). Although the episodes of bradycardia, atrial arrhythmia, bundle branch block and the change in the ST-T tended to decrease post-treatment, the difference did not achieve statistical significance in this small sample (Table II).

## Discussion

Despite the therapeutic efficacy suggested by previous studies (9,11), IVIG therapy for AFM has been rarely reported. In this study, IVIG therapy improved the LVEF and reduced the LVDD compared with the control group. Furthermore, compared with the baseline values, the LA, LVDD, LVDS, RV and the episodes of VT/VF and AVB were improved at the post-treatment time-point in the IVIG group.

The damage to the myocardium in AFM may be mediated by predominantly immunological mechanisms rather than by the direct effect of viral infection and replication (13,14). Previous studies have indicated the therapeutic effects of the IVIG in acute myocarditis (11,15). Weller *et al*(11) observed that mouse polyclonal immunoglobulin minimized myocardial damage in Balb/c male mice infected intraperitoneally with coxsackievirus B3 when administered 24 or 48 h subsequent to infection (11). In another murine model of viral myocarditis, IVIG administration reduced the extent of myocardial necrosis or interstitial fibrosis and improved ventricular remodeling (15). In addition, certain clinical trials have indicated that IVIG may be beneficial in acute myocarditis. Drucker *et al*(9) suggested that the use of high-dose IVIG for the treatment of acute myocarditis was associated with the improved recovery of left ventricular function and with a improved survival rate during the first year following presentation (9). In patients with new-onset dilated cardiomyopathy treated with high-dose IVIG, LVEF improved by 17 EF units (16). IVIG induced a significant increase in LVEF from 26±2 to 31±3% in 40 patients with symptomatic chronic congestive heart failure (CHF) and LVEF of <40% (17). A favorable clinical response was also observed in 10 case studies (18–27) and two case series (28,29). However, according to a recent systematic review (30), certain studies (31,32) showed no benefit of IVIG. A randomized controlled trial (RCT) suggested that IVIG did not augment the improvement in LVEF for 62 patients with recent-onset dilated cardiomyopathy. This RCT showed no benefit with respect to cardiac function, functional outcome or event-free survival (33).

Certain studies have suggested the mechanism underlying the effect of high-dose IVIG in acute myocarditis (17,34–36). IVIG induced a marked rise in plasma levels of the anti-inflammatory mediators interleukin (IL)-10, IL-1 receptor antagonist and soluble tumor necrosis factor receptors. Furthermore, levels of N-terminal pro-atrial natriuretic peptide continued to decrease toward the end of the study during IVIG therapy (17). IVIG decreased cardiac inflammation and downregulated proinflammatory cytokines that have direct negative inotropic effects (34–36).

IVIG decreases the incidence of VT/VF and AVB in AFM in the present study. The combination of arrhythmias, including VT/VF and AVB, and heart failure presents a serious challenge in the management of AFM (1–4). The arrhythmias were resolved during the convalescent and remote phases among the surviving patients, and no atrial or ventricular arrhythmias were induced by the programmed stimulation (4).

In conclusion, this study suggested that IVIG for the treatment of AFM may be associated with improved recovery of left ventricular function and a reduction in the episodes of fulminant arrhythmias.

## Figures and Tables

**Table I:** Initial demographic and clinical characteristics of 58 patients with AFM.

Variable	IVIG group (n=32)	Control group (n=26)	P-value
Age (years)^a^	30.6±14.1	30.0±16.6	0.905
Male^b^	15/32 (47)	13/26 (50)	0.813
NYHA classification^b^			0.144
Class I	0/32 (0)	0/26 (0)	
Class II	8/32 (25)	6/26 (23)	
Class III	13/32 (41)	11/26 (42)	
Class IV	11/32 (34)	9/26 (35)	
Echocardiography^a^			
Left ventricular ejection fraction (%)	45.3±15.2	43.5±19.6	0.703
Diameter of left atrium (mm)	30.3±5.3	31.5±7.3	0.711
Diameter of right atrium (mm)	43.0±8.7	46.1±9.0	0.211
Left ventricular diastolic diameter (mm)	47.1±7.3	50.1±9.3	0.197
Left ventricular systolic diameter (mm)	35.3±8.9	37.9±12.2	0.543
Diameter of right ventricle (mm)	50.9±7.7	50.5±13.7	0.898
Laboratory tests^a^			
A-hydroxybutyrate dehydrogenase (U/l)	959.2±1167.6	641.8±486.8	0.585
Lactate dehydrogenase (U/l)	1707.7±3021.5	923.6±851.9	0.632
Creatine kinase (U/l)	2228.3±5271.8	889.1±825.7	0.179
Creatine kinase-MB (U/l)	40.4±56.2	28.8±23.9	0.454
Troponin (ng/ml)	10.2±21.8	2.2±2.7	0.789
Brain natriuretic peptide (pg/ml)	12746.8±13620.1	4690.6±8780.7	0.117
C-reactive protein (mg/l)	46.5±51.2	34.9±31.7	0.480
Erythrocyte sedimentation rate (mm/h)	20.0±16.5	19.7±18.8	0.969
Complications^b^			
Pulmonary edema	13/32 (41)	7/26 (27)	0.428
Cardiogenic shock	12/32 (38)	4/26 (15)	0.061
Multiple Organ Dysfunction Syndrome	8/32 (25)	2/26 (8)	0.166
Renal failure	8/32 (25)	1/26 (4)	0.065
Cardiac arrest	1/32 (3)	2/26 (8)	0.853
Aspen syndrome	2/32 (6)	4/26 (15)	0.482
Therapies^b^			
Vitamin C	30/32 (94)	23/26 (88)	0.808
Glucocorticoid	28/30 (93)^c^	18/19 (95)^c^	0.088
Intra-aortic balloon pumps	10/30 (33)^c^	3/19 (16)^c^	0.073
Pacemaker	15/30 (50)^c^	8/19 (42)^c^	0.212
Mortality^b^	2/32 (6)	7/26 (27)	0.072

a Values are presented as the mean ± standard deviation, the data of left ventricle and left atrium were measured on parasternal long-axis view, and the data of right ventricle and right atrium were measured on apical four-chamber view.

b Values are presented as the number of patients (%).

c Values are presented as the number of remaining patients following mortalities.

IVIG, intravenous immunoglobulin; AFM, acute fulminant myocarditis; NYHA, New York Heart Association.

**Table II:** Incidence of arrhythmia and the change in ST-T in the IVIG and control groups pre- and post-treatment.

	IVIG group (n=32)							Control group (n=26)						
		
Time-point	Bradycardia	AVB	VT/VF	Atrial arrhythmia	Ventricular arrhythmia	BBB	ST-T change	Bradycardia	AVB	VT/VF	Atrial arrhythmia	Ventricular arrhythmia	BBB	ST-T change
Baseline n (%)	17 (53.1)	17 (53.1)	8 (25.0)	6 (18.8)	10 (31.3)	17 (53.1)	25 (78.1)	11 (42.3)	8 (30.8)	3 (11.5)	2 (7.7)	6 (23.1)	6 (23.1)	15 (57.7)
Post-treatment n (%)	4 (12.5)	3^a^ (9.4)	1^a^ (3.1)	1 (3.1)	2 (6.3)	6 (18.8)	12 (37.5)	3 (11.5)	4 (15.4)	3 (11.5)	0 (0.0)	3 (11.5)	4 (15.4)	7 (26.9)

a P<0.05 vs. baseline value.

AVB, atrioventricular block; VT, ventricular tachycardia; VF, ventricular fibrillation; BBB, bundle branch block; IVIG, intravenous immunoglobulin.
